# NMR and X-ray analysis of structural additivity in metal binding site-swapped hybrids of rubredoxin

**DOI:** 10.1186/1472-6807-7-81

**Published:** 2007-12-05

**Authors:** David M LeMaster, Janet S Anderson, Limin Wang, Yi Guo, Hongmin Li, Griselda Hernández

**Affiliations:** 1 Wadsworth Center, New York State Department of Health and Department of Biomedical Sciences, School of Public Health, University at Albany – SUNY, Empire State Plaza, Albany, New York 12201, USA; 2 Department of Chemistry, Union College, Schenectady, New York 12308, USA

## Abstract

**Background:**

Chimeric hybrids derived from the rubredoxins of *Pyrococcus furiosus *(*Pf*) and *Clostridium pasteurianum *(*Cp*) provide a robust system for the characterization of protein conformational stability and dynamics in a differential mode. Interchange of the seven nonconserved residues of the metal binding site between the *Pf *and Cp rubredoxins yields a complementary pair of hybrids, for which the sum of the thermodynamic stabilities is equal to the sum for the parental proteins. Furthermore, the increase in amide hydrogen exchange rates for the hyperthermophile-derived metal binding site hybrid is faithfully mirrored by a corresponding decrease for the complementary hybrid that is derived from the less thermostable rubredoxin, indicating a degree of additivity in the conformational fluctuations that underlie these exchange reactions.

**Results:**

Initial NMR studies indicated that the structures of the two complementary hybrids closely resemble "cut-and-paste" models derived from the parental *Pf *and *Cp *rubredoxins. This protein system offers a robust opportunity to characterize differences in solution structure, permitting the quantitative NMR chemical shift and NOE peak intensity data to be analyzed without recourse to the conventional conversion of experimental NOE peak intensities into distance restraints. The intensities for 1573 of the 1652 well-resolved NOE crosspeaks from the hybrid rubredoxins were statistically indistinguishable from the intensities of the corresponding parental crosspeaks, to within the baseplane noise level of these high sensitivity data sets. The differences in intensity for the remaining 79 NOE crosspeaks were directly ascribable to localized dynamical processes. Subsequent X-ray analysis of the metal binding site-swapped hybrids, to resolution limits of 0.79 Å and 1.04 Å, demonstrated that the backbone and sidechain heavy atoms in the NMR-derived structures lie within the range of structural variability exhibited among the individual molecules in the crystallographic asymmetric unit (~0.3 Å), indicating consistency with the "cut-and-paste" structuring of the hybrid rubredoxins in both crystal and solution.

**Conclusion:**

Each of the significant energetic interactions in the metal binding site-swapped hybrids appears to exhibit a 1-to-1 correspondence with the interactions present in the corresponding parental rubredoxin structure, thus providing a structural basis for the observed additivity in conformational stability and dynamics. The congruence of these X-ray and NMR experimental data offers additional support for the interpretation that the conventional treatment of NOE distance restraints contributes substantially to the systematic differences that are commonly reported between NMR- and X-ray-derived protein structures.

## Background

Mutational analyses are increasingly being utilized to elucidate the structural basis of concerted conformational transitions, as well as to address the related question of how conformational dynamics propagate through a protein molecule [1-4]. Enzymes that undergo concerted active-site transitions which occur within the timeframe of substrate turnover provide evidence for the role of conformational fluctuations in catalysis [5-8]. Mutations that disrupt such collective active-site transitions result in reduced catalysis [9,10]. However, more detailed analysis of these conformational transitions must confront the complication that most mutations introduce interactions that are not present in the parental protein structure. As a result, it is generally ambiguous whether the altered conformational dynamics directly reflect the properties of the parental protein, or whether they reflect the novel interactions that are introduced by the mutation.

The rubredoxin from the hyperthermophilic archaeon *Pyrococcus furiosus *(*Pf*) is the most thermostable monomeric protein that is known to reversibly unfold [11]. A structure design algorithm [12] was applied to the rubredoxins from *Pyrococcus furiosus *and the mesophilic bacterium *Clostridium pasteurianum *(*Cp*) to generate chimeric proteins in which the predicted hybridization interface can preserve each of the interactions in the parental structures. Interchange of the seven nonconserved residues in the metal binding site region increases the T_m _of *Cp *rubredoxin by 13°, while the complementary exchange yields an equivalent decrease in T_m _for the parental hyperthermophilic protein. The differential free energies of stability, between the parental and metal binding site-swapped hybrid rubredoxins, account for 39% of the differential thermodynamic stability between the two parental rubredoxins [13]. These rubredoxin chimeras appear to constitute the first structurally designed pair of hybrids that exhibit full thermodynamic additivity upon exchange of a cluster of mutually interacting residues which define a substantial hybridization interface across the interior of a protein domain.

The additivity in thermodynamic stability for each rubredoxin hybrid is consistent with the hybridization interfaces of these proteins being composed of a complementary sum of parental-like interactions. In addition to its utility in characterizing the structural basis of differential thermodynamic stability, a set of protein hybrids that preserves the full set of parental-like interactions offers a highly promising system for analysis of conformational dynamics. It is only under the condition of such a complementarity in the "ground state" energetics that an analogous additivity in the "excited state" conformational dynamics can be anticipated.

The changes in amide hydrogen exchange rates that result from substitution of the *Cp *metal binding site residues into the hyperthermophile rubredoxin are faithfully mirrored by the symmetric set of differential rates that arise from the complementary residue cluster exchange. This behavior applies throughout the sequence, affecting even structurally buried residues for which the exchange rates are retarded well beyond a million-fold from the simple model peptide rates. As a result, the conformational transitions that must precede these hydrogen exchange reactions exhibit an additivity in their dynamics for the two complementary hybrids, relative to the dynamics of the parental *Cp *and *Pf *rubredoxins [14]. The non-mutated residues that exhibit more than a 3-fold change in differential exchange rate upon interchange of the metal binding site residues are almost exclusively structurally buried. The sidechains of these affected residues form a connected set of interactions that penetrates across much of the protein interior.

The pair of metal binding site-swapped hybrids of *Pf *and *Cp *rubredoxin were designed on the basis of their potential to form structures that correspond to a "cut-and-paste" of the parental protein structures. The observed additivity in both thermodynamic stability and conformational dynamics is consistent with the detailed preservation of the energetic interactions predicted for such a "cut-and-paste" structure. The first direct evidence for a corresponding additivity in structure came from 2D ^1^H-^15^N correlation experiments which demonstrated that the spectra of the hybrids are well represented by a simple combination of the parental protein spectra [13]. In contrast, when the resonances from the 31 sequence-conserved residues of the parental *Pf *and *Cp *rubredoxins were compared against one another, substantially larger differences in chemical shift were observed. As a result, it can be anticipated that the deviations from a structural additivity for the metal binding site-swapped hybrids would be smaller than the 0.63 Å rmsd value observed between the backbones of the parental *Pf *and *Cp *rubredoxin structures.

Whether conventional NMR structure determination is adequate for this level of structural discrimination is a point of active debate. Based on analysis of the relative geometric quality of protein structures derived from NMR data and from X-ray data, Vuister and colleagues [15] have argued that the quality of NMR structures can be best compared to that of X-ray structures at ~4 Å resolution. This comparison is somewhat problematic to assess quantitatively, since only 0.2% of all protein X-ray structures in the Protein Data Bank [16] have reported resolution limits of 4 Å or above. However, further support for such an interpretation comes from a recent structural analysis by Montelione and coworkers [17]. Among the structural quality evaluation tools that they considered, these authors identified the Procheck [18] dihedral angle G-factor and MolProbity [19] score as the most sensitive measures of X-ray crystal structure accuracy. A set of 252 X-ray structures of resolution ≤ 1.8 Å were used to define the high resolution reference for the Z-score analysis of 587 NMR protein structures. Mean Z-scores of -4.82 (Procheck, including all dihedrals) and -8.22 (MolProbity) were obtained for these NMR structures, as compared to mean Z-scores of -2.46 and -4.51, respectively, for the low resolution X-ray structure set (resolution > 2.50 Å and ≤ 3.50 Å). According to these measures, the amount by which the NMR structures are less accurate than the low resolution X-ray structures is effectively equivalent to the difference in accuracy between the high and low resolution X-ray data sets.

To date, the average reported precision of NMR structures is 0.6 Å for the well-ordered backbone atoms [20]. In contrast, a recent analysis of 60 nonhomologous proteins [21], for which the X-ray and NMR structures have been reported to exhibit no large-scale structural differences, found that the median backbone rmsd value between the corresponding structures was 1.6 Å. Particularly germane to our present investigation is the fact that these 60 NMR structures exhibit residue contact densities that are 15% higher than the densities in the corresponding X-ray structures for distances near the van der Waals contact limit as well as for distances near 6 Å [21], at each extremum of the conventional NMR distance restraint boundaries. Such a systematic difference in the local packing interactions may help to account for the failure of NMR structures to be accurately distinguishable from decoy structures by native structure recognition algorithms that have been optimized with X-ray structural coordinates [22].

Differences between the crystalline environment and the solution state have often been invoked to explain why corresponding X-ray and NMR-derived protein structures generally differ from one another by substantially more than the estimated uncertainties of either structure. However, arguments from the differences in crystal and solution conditions would appear inadequate to explain the spatially localized increase in relative packing densities reported for the NMR solution structures [21]. Such a systematic biasing of the packing densities at each extremum of the standard NMR distance restraint boundaries strongly suggests that the conventional estimation of distance restraints from the experimental NOE data and the subsequent processing of these restraints contribute significantly to the deviations reported between X-ray and NMR protein structures.

If the structures of the metal binding site-swapped rubredoxins do form as designed, for every NOE crosspeak in the spectra of the hybrid rubredoxins there exists a 1-to-1 correspondence with an analogous NOE crosspeak in the parental spectra. As a result, each NOE crosspeak from the hybrid rubredoxins can be directly compared to the analogous crosspeak from the parental *Cp *or *Pf *protein to see whether the difference in peak intensity significantly exceeds the noise level of the experimental data. In the absence of such a difference in NOE peak intensity, we may conclude that the structures of the rubredoxin hybrids correspond to a "cut-and-paste" of the parental *Cp *and *Pf *rubredoxins to within the information content of the raw NOE data. Conversely, any systematic differences seen in the corresponding NOE crosspeaks will indicate either a change in the interatomic distances for the surrounding protons or else a change in the local conformational/chemical dynamics. Ultra-high resolution X-ray diffraction analysis provides a powerful complementary demonstration of the degree to which the structural additivity observed in the solution state is preserved upon crystallization.

## Results

### Differential structural analysis of complementary protein hybrids in solution

In favorable cases, the degree to which the structures for a complementary pair of chimeric protein hybrids correspond to a simple sum of the parental protein structures can be characterized via a differential analysis of the chemical shifts and NOE intensities. Although quantitative prediction of chemical shifts presents a major ongoing challenge, the exquisite sensitivity of these shifts to the local environment can provide an excellent criterion by which to identify the boundary junctions to be used in construction of an initial protein model and, subsequently, for qualitative assessment of that model. Quantitative NOE peak intensities provide an independent criterion against which the consistency of the structure of the hybrid proteins with the corresponding "cut-and-paste" models can be assessed. The more straightforward dependencies of the NOE cross relaxation rate upon the local geometry and internal motion provide a basis for structural interpretation of the intensity differences that occur between the NOE crosspeaks of the hybrid and the corresponding parental spectra as predicted from the 'cut-and-paste" model.

### Differential chemical shift analysis of the metal binding site-swapped rubredoxin hybrids

**Figure 1:**
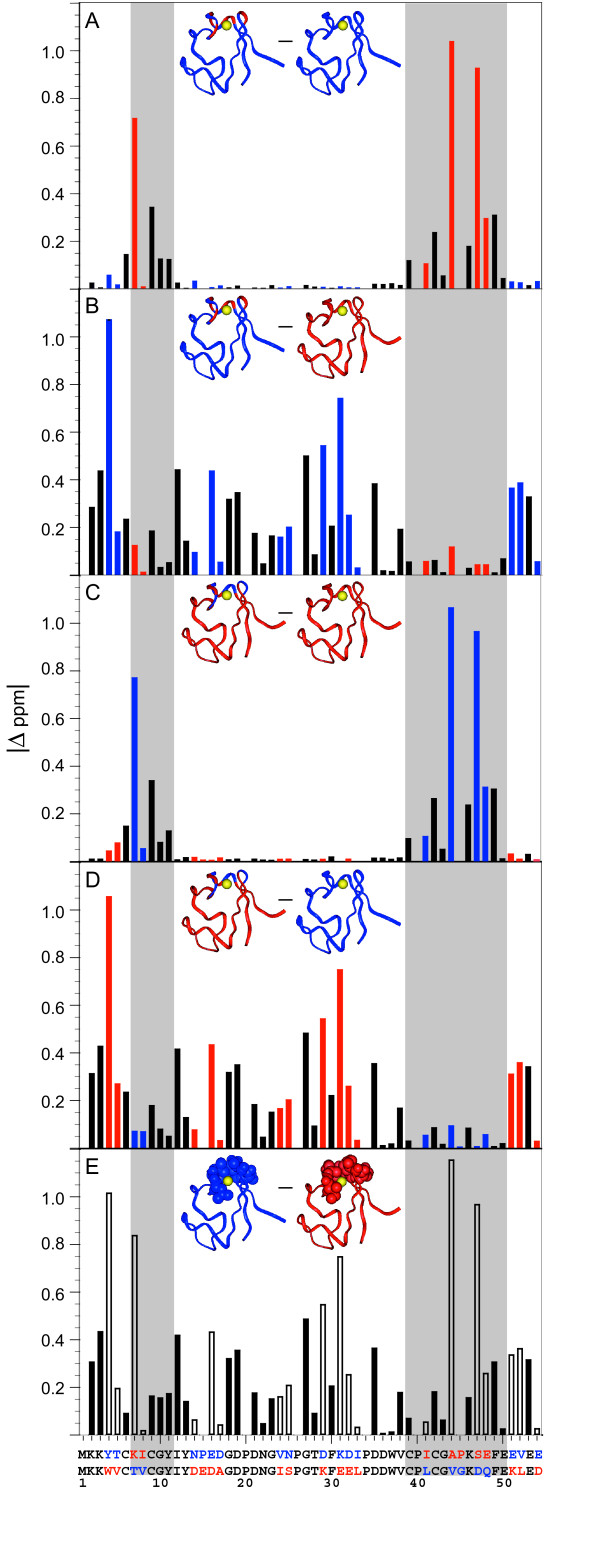
**Absolute differences in the amide ^1^H chemical shifts among pairs of parental and metal binding site-swapped rubredoxins**. The chemical shift differences between each hybrid and each parental protein are illustrated in panels A to D. Panel E shows the corresponding differences between the parental *Cp *and *Pf *A2K rubredoxins. In this last panel, the seven nonconserved metal binding site residues are highlighted in CPK representation for each parental protein. For the bars and the protein figures inserted in each panel, as well as in the sequence alignment at the bottom, red denotes residues derived from the hyperthermophilic *Pf *A2K rubredoxin sequence, while blue denotes residues derived from the mesophilic *Cp *sequence. Sequence-conserved residues are indicated with solid black bars. The gray background indicates the sequence segments that have been interchanged so as to generate the models for the metal binding site-swapped hybrids. The mutations for the hyperthermophile protein in these segments consist of T7K, V8I, L41I, V44A, G45P, D47S and Q48E.

In the analysis of whether the two metal binding site-swapped hybrids can each be accurately represented as a sum of the parental *Pf *and *Cp *rubredoxin structures, the critical decisions in the initial model construction involve those residues that are conserved in each sequence but may differ in their local conformation, due to interactions with nonconserved residues. The chemical shifts of the sequence conserved residues serve to identify which parental rubredoxin presents a local environment for each residue which is most similar to that for the analogous residue in each of the hybrid proteins. The absolute differences in the amide chemical shifts observed between each hybrid and each parental rubredoxin, as well as between the parental proteins themselves, are given in Figure 1. An Ala 2 to Lys variant of *Pf *rubredoxin (*Pf *A2K) was utilized as the hyperthermophile reference, due to its efficient N-terminal processing during *Escherichia coli *expression [23] and its increased similarity with the N-terminal interactions of the *Cp *protein. In every case for which the amide ^1^H chemical shift of a residue in a given hybrid is more similar to that of one parent, the corresponding shift for the other hybrid is more similar to that of the other parent, indicating a clear complementarity in the differential chemical shifts of the two hybrid rubredoxins. Interchange of the two segments containing residues 7 to 11 and 39 to 50 results in the minimum differential chemical shift behavior for both the amide ^1^H and ^15^N resonances.

**Figure 2:**
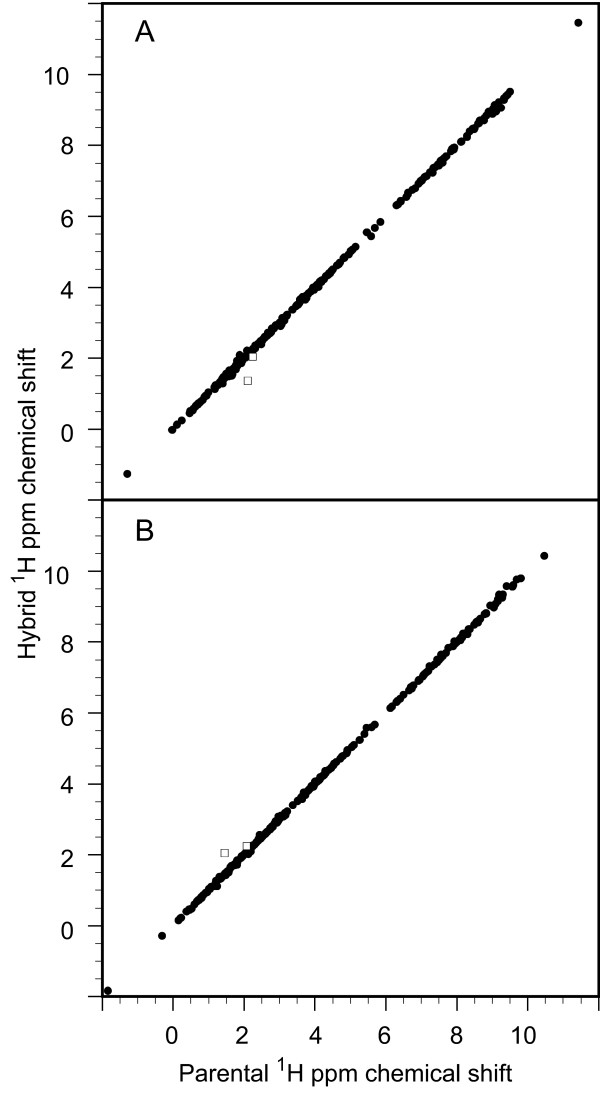
**Correlations among all hybrid and parental rubredoxin ^1^H chemical shifts**. The amide chemical shift data of Figure 1 were used to assign each individual residue of the metal binding site-swapped hybrids to a particular parent. Panel A illustrates the comparison to parental chemical shifts for the metal binding site-swapped *Cp *rubredoxin, while the analogous ^1^H data for the complementary metal binding site-swapped *Pf *A2K rubredoxin hybrid are shown in panel B. Only the H^ε ^resonances of Lys 46 (open diamonds) deviate substantially from equivalence.

Based on this minimum differential amide chemical shift criterion, when all of the mainchain and sidechain ^1^H resonances of each hybrid rubredoxin residue are assigned to the corresponding parent, an excellent correlation is observed (Figure 2). The rmsd values for the differential ^1^H chemical shifts of the sequence-conserved residues between each of the two metal binding site-swapped hybrids and its corresponding parental rubredoxin are 0.043 and 0.042 ppm (Table 1). In contrast, when these same resonances are compared to the "opposite" parental rubredoxin or compared between the parental *Pf *and *Cp *rubredoxins, 4-fold larger rmsd values are obtained. These larger rmsd values are similar to those obtained from the correlation between pairs of homologous proteins having the same level of sequence identity (0.328 ppm for H^N^, 0.164 ppm for H^α ^and 0.246 ppm for H^β ^at a sequence identity level of 59%) [24].

**Table 1:** Differential chemical shifts between pairs of hybrid, *Cp *and *Pf *A2K rubredoxins.

	^1^HN	^15^N	^1^H(all)	^1^HN(con)^a^	^15^N(con)	^1^H(con)
*Pf *metal site-swapped *Cp *hybrid-parent^b^	0.046	0.279	0.042	0.049	0.318	0.043
*Pf *metal site-swapped *Cp *hybrid-non-parent^c^	-	-	-	0.301	1.872	0.191
*Cp *metal site-swapped *Pf *hybrid-parent	0.044	0.264	0.038	0.049	0.277	0.042
*Cp *metal site-swapped *Pf *hybrid-non-parent	-	-	-	0.334	1.871	0.175
*Cp *- *Pf *A2K	-	-	-	0.286	1.785	0.181

a. Resonances from residues that are conserved between the parental sequences.

b. Residue-specific parental type assignment based on minimal amide chemical shift difference.

c. *Cp *or *Pf *A2K resonance not assigned to the hybrid resonance based on amide chemical shift.

The close correlation between the chemical shifts of the sequence-conserved residues of the rubredoxin hybrids and the corresponding parental protein chemical shifts indicates that this analysis is sensitive to variations in local conformation which are subtle, relative to the structural differences between the parental proteins. The correlation between the parental and hybrid chemical shifts strongly supports the validity of the proposed structural partitioning based on the differential amide chemical shift data.

The striking correlation in the ^1^H shifts illustrated in Figure 2 indicates that, with one exception, the sidechain resonances for every residue in each hybrid protein closely mimic the corresponding sidechain resonances of the parental rubredoxin that is assigned to that hybrid protein on the basis of the backbone amide shifts. Hence, in this protein system, the parsing of cleavage boundaries in forming the "cut-and-paste" models can be effectively done at the residue level, rather than requiring the partitioning of atoms within an individual residue. Only the chemical shifts of Lys 46 H^ε ^deviate substantially between the hybrids and corresponding parental rubredoxins, as defined by the amide shift comparison. The hybrid-parent chemical shift values for Lys 46 H^ε ^are displaced symmetrically away from the diagonal in panels A and B of Figure 2, indicating that structural correlation with the "opposite" parent is more appropriate for these protons. The structural significance of this reversed chemical shift correlation for the Lys 46 sidechain will be considered in more detail below.

### Differential analysis of hybrid rubredoxin NOE crosspeaks

A differential NOE analysis can utilize either peak heights or peak volumes. NOE volumes are generally used, since they are nominally proportional to the interproton cross relaxation rates, which in turn depend upon the interproton distances. The corresponding peak heights exhibit additional dependencies on local structure and conformational dynamics. However, peak height measurements generally yield higher signal-to-noise values than do peak volumes when a given resonance is monitored among a series of related spectra, as illustrated in spin relaxation studies. The relative statistical advantage of the peak height measurement becomes more enhanced as the intrinsic sensitivity of the experiment decreases, as in the case of the often weak NOE crosspeaks. An additional practical advantage of peak height-based analysis occurs when quantitative comparisons are made among differing resonances, since peak height estimates are generally less sensitive to the selected footprint of the resonance than are the corresponding volume estimates. If the set of analogous parental and hybrid NOE crosspeaks that define the structure of a local region exhibits statistically equivalent peak heights, this provides strong support for a highly similar structure within that region of the proteins. When such equivalent peak heights are not observed, the structural and dynamical effects that underlie the deviations must be resolved by additional analysis.

**Figure 3:**
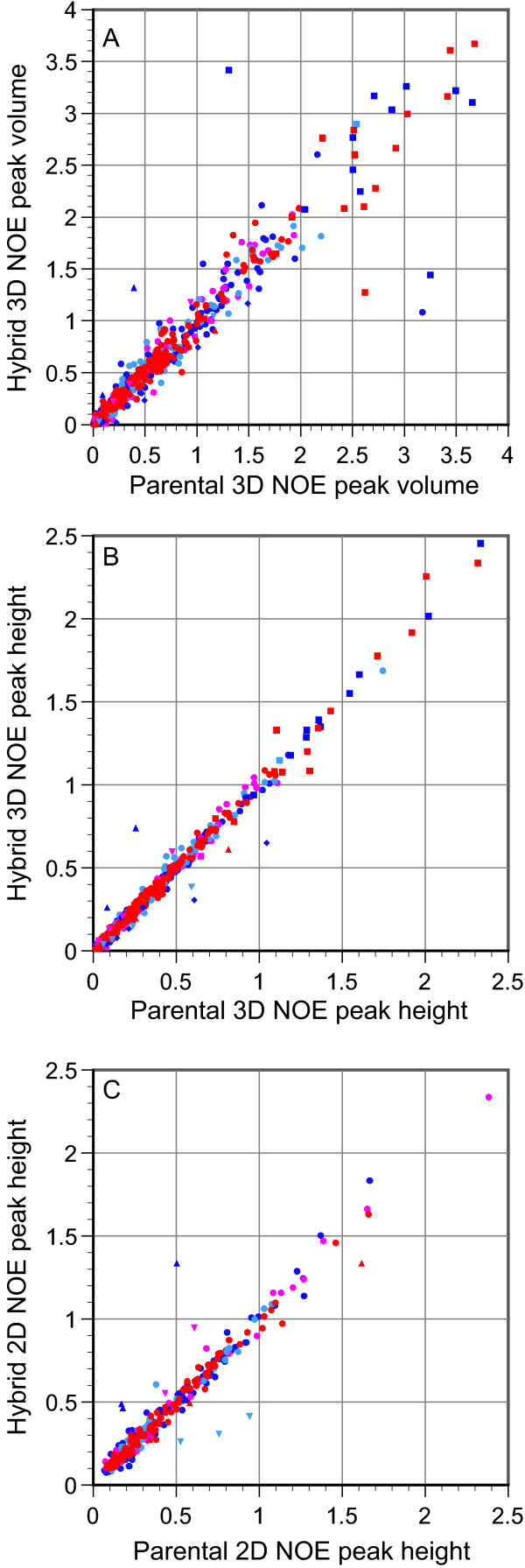
**Comparison between NOE crosspeaks from hybrid and parental *Cp *and *Pf *A2K rubredoxins**. Hybrid rubredoxin NOE crosspeaks between protons assigned to a common parent are compared to the corresponding parental peaks. Normalized volumes (panel A) and heights (panel B) from the 3D ^1^H-^15^N-^1^H 3D NOESY data are given for 1045 pairs of hybrid and parental NOE peaks, while panel C provides analogous peak height data from 2D ^15^N suppressed ^1^H-^1^H NOESY experiments (365 NOE pairs). The hybrid-parent type for each pair of NOE peaks is indicated in red (metal binding site-swapped *Pf *A2K and *Pf *A2K), magenta (metal binding site-swapped *Cp *and *Pf *A2K), light blue (metal binding site-swapped *Pf *A2K and *Cp*), and dark blue (metal binding site-swapped *Cp *and *Cp*). Individual symbols denote NOE crosspeaks involving Lys 2 H^N ^(◆), Thr 28 H^γ1 ^(▲), Phe 49 H^δ ^(▼) and sequential H^α^-H^N ^connectivities for conformationally extended residues (■). The intraresidue Trp 37 H^ε3^-H^ζ3 ^and Trp 37 H^δ1^-H^ε1 ^crosspeaks were used as intensity references for the 2D and 3D NOE data, respectively.

Figure 3 illustrates the relative peak volumes (panel A) and peak heights (panel B) of well-resolved crosspeaks for 1045 hybrid-parent comparisons from 3D ^1^H-^15^N-^1^H NOESY spectra. This figure only includes the crosspeaks for which both protons in the metal binding site-swapped hybrids are assigned to the same parental (*Cp *or *Pf *A2K) rubredoxin structural type, based on the amide chemical shift analysis of Figure 1. The NOE crosspeaks involving Lys 2 H^N^, Thr 28 H^γ1 ^and Phe 49 H^δ^, as well as the crosspeaks from sequential H^α^-H^N ^connectivities in conformationally extended residues are indicated by characteristic symbols to highlight their increased deviations from the diagonal. With the exception of these highlighted crosspeaks, the rmsd for the relative peak heights of the corresponding hybrid and parental NOE peaks is 0.022, while the relative rmsd value for the peak volume comparison is 3-fold higher. An rmsd value of 0.043 is obtained for the analogous comparison of 365 hybrid-parental NOE pairs from 2D ^15^N suppressed ^1^H-^1^H NOESY spectra (panel C).

The spread of the data around the diagonal in Figure 3B is largely independent of the absolute peak height value, consistent with the baseplane noise providing the primary source of experimental uncertainty. Comparison between the peak height of the intraresidue Trp 37 H^δ1^-H^ε1 ^crosspeak, used to normalize the 3D NOE intensities, and the baseplane noise level of the various NOESY spectra indicates that the resultant contribution of baseplane noise to the peak height uncertainty is within a factor of 2 of the dispersion around the diagonal. Hence, the structural differences for the corresponding hybrid and parental interactions that underlie the vast majority of the NOE crosspeaks summarized in this figure do not yield variations in peak height that are significantly above the intrinsic noise level of the data.

The structural discrimination offered by our differential NOE analysis can be estimated from the comparison of crosspeaks between pairs of protons on sequence-conserved residues in the parental *Cp *and *Pf *A2K rubredoxins. If the same set of resonances as in Figure 3 are filtered out, an rmsd of 0.053 is obtained when the parental 3D NOE crosspeaks are compared. This dispersion is nearly 2 1/2-fold greater than for the matched hybrid-parental NOE data of Figure 3B. Hence, analogous to the differential chemical shift analysis, this differential NOE comparison provides a level of discrimination that is subtle on the scale of the structural variations between sequence-conserved residues in the two parental rubredoxins.

In Figure 3, the NOE crosspeaks arising from each set of connectivities for the Lys 2 H^N^, Thr 28 H^γ1 ^and Phe 49 H^δ ^protons deviate from the diagonals by approximately proportional amounts. Due to rapid hydrogen exchange of the Lys 2 amide proton, no NOE crosspeaks are observed for either *Pf *A2K rubredoxin or the metal binding site-swapped *Pf *rubredoxin hybrid at pH 6.0 and 23°C. Relaxation-compensated CLEANEX-PM experiments [25,26] on *Cp *rubredoxin and the metal binding site-swapped *Cp *rubredoxin hybrid indicate hydrogen exchange rates under these conditions of 1.5 and 7.0 s^-1^, respectively, providing an explanation of the proportionately smaller heights for each of the 10 Lys 2 H^N ^NOE peaks of this hybrid, relative to those of the parental *Cp *rubredoxin. Similarly, the markedly broader resonance of Thr 28 H^γ1 ^in *Cp *rubredoxin, relative to the corresponding linewidth in the other three proteins, is consistent with more rapid hydrogen exchange for this hydroxyl proton. The 14 pairs of Thr 28 H^γ1 ^NOE hybrid-parent peak heights vary accordingly. Under the pH and temperature conditions used, hydroxyl proton resonances are commonly not observed, due to rapid chemical exchange with the bulk water resonance [27].

Each of the 21 crosspeaks involving Phe 49 H^δ ^is consistent with a more modest linebroadening of this resonance in the metal binding site-swapped *Cp *hybrid and the parental *Cp *rubredoxin, relative to the other two proteins. In this case, linebroadening due to chemical exchange dynamics must arise from the interchange of the ring protons between differing chemical shift environments in the μs-ms timeframe, most likely due to flipping around the 2-fold symmetry axis of the phenyl group at a rate near the fast exchange limit.

In an extended backbone conformation, the sequential H^α^-H^N ^protons are within van der Waals contact distance. This class of NOE crosspeaks is known to be particularly sensitive to the conformational dynamics effects that arise from asymmetry between the radial and angular components of the interproton vector fluctuations [28]. Such heightened sensitivity to asymmetrical fluctuations may account for the increased variability of the peak heights for these crosspeaks (Figure 3B).

It must be emphasized that the marked similarity in peak height for the vast majority of analogous hybrid and parental rubredoxin NOE crosspeaks does not imply an absence of absolute intensity variations due to internal motion for these atoms. Rather, it indicates that in most cases the NOE-effective conformational dynamics of the parental *Cp *and *Pf *A2K rubredoxins are faithfully partitioned into the two complementary hybrid structures. This partitioning of dynamics can be illustrated by the aromatic ring of Tyr 13, for which the phenolic hydroxyl proton has 20 and 18 well-resolved NOE crosspeaks in the *Cp *and *Pf *A2K rubredoxins, respectively. However, in contrast to the 31 NOE crosspeaks for the Tyr 13 H^δ ^and H^ε ^resonances of *Pf *A2K rubredoxin, there are only three low intensity NOE peaks for the analogous ring protons of *Cp *rubredoxin, reflecting resonance broadening due to the ring flip dynamics of this sidechain near room temperature. While the Tyr 13 NOE crosspeaks of the metal binding site-swapped *Pf *hybrid closely mimic those of *Pf *A2K rubredoxin, the Tyr 13 ring protons of the metal binding site-swapped *Cp *hybrid have the same three ring proton NOE crosspeaks as observed in *Cp *rubredoxin and three additional weak crosspeaks that have a maximal normalized amplitude of only 0.035.

### NOE analysis across the hybridization interface

When an NOE crosspeak in the rubredoxin hybrids arises from two protons that lie on opposite faces of the hybridization interface, one proton will be assigned to the *Cp *parental structure type, while the other proton will be of the *Pf *rubredoxin structure type. Hence, these cross-interface NOE crosspeaks were not included in the hybrid-parent NOE intensity comparisons of Figure 3. In the initial design of the metal binding site-swapped hybrids, for every predicted cross-interface NOE interaction at least one of the two protons is stereochemically equivalent for each of the parental rubredoxins [12]. The case in which both protons are stereochemically equivalent in each parental rubredoxin is illustrated by the crosspeak between Phe 30 H^ζ ^and Lys 46 H^α^. These NOE crosspeaks have normalized peak heights of 0.472 in the parental *Pf *A2K rubredoxin, 0.483 in the metal binding site-swapped *Pf *hybrid, 0.388 in *Cp *rubredoxin and 0.325 in the metal binding site-swapped *Cp *hybrid. This NOE interaction in the metal binding site-swapped *Pf *hybrid most closely mimics that of the parental *Pf *A2K rubredoxin, while this interaction in the other hybrid is more similar to that of the parental *Cp *protein. This pattern is reverse of that predicted from the residue-based parental type assignments utilizing the amide chemical shift data of Figure 1. Hence, the sidechain interactions of Lys 46 appear to follow the parental type of the protein core (Figure 2) rather than the parental type of the metal binding site residues which determine the chemical shift behavior of the Lys 46 amide resonances.

**Figure 4:**
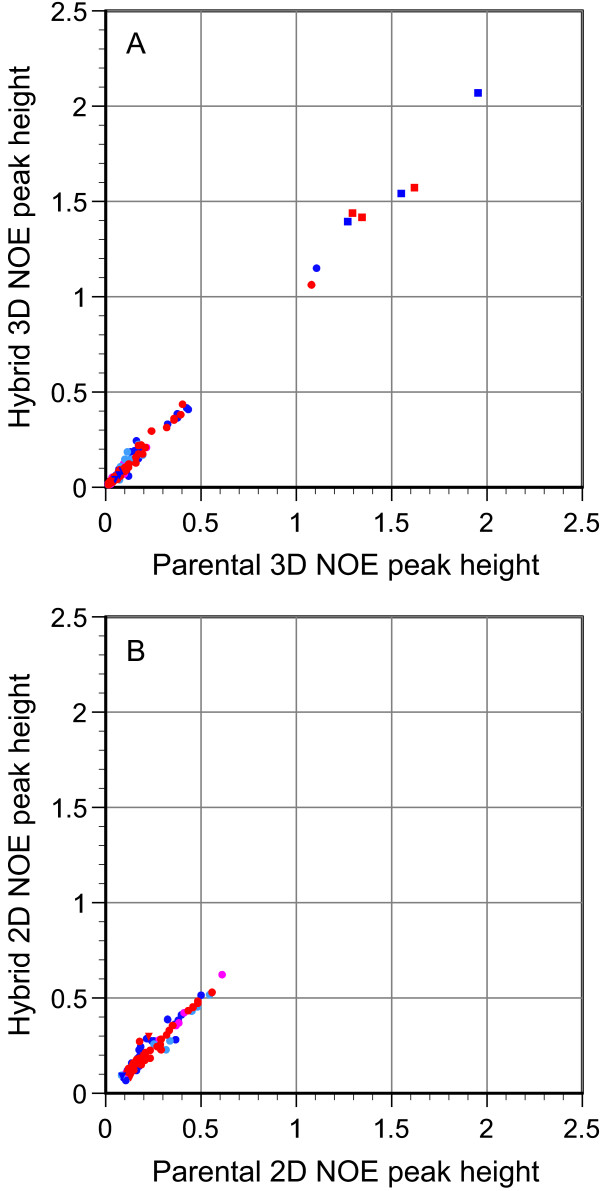
**Peak height comparison for NOE interactions that span the hybridization interface**. Metal binding site-swapped hybrid NOE peak heights for which the two protons do not correlate to the same parental rubredoxin, based on the minimum amide chemical shift criterion, are compared to the analogous parental NOE peaks, based on the similarity in peak height, for 163 3D NOE pairs (panel A) and 79 2D NOE pairs (panel B). Color-coding and symbols are as defined in Figure 3.

Each metal binding site-swapped hybrid NOE crosspeak between two structurally conserved protons that spans the hybridization interface can be correlated with the more similar of the two analogous parental NOE crosspeaks, while cross-interfacial NOE peaks between a conserved proton and a nonconserved proton can be compared to the corresponding parental rubredoxin peak. Peak height comparisons for the 79 2D and 163 3D cross-interfacial NOE peaks (Figure 4) exhibit a dispersion similar to that for the hybrid crosspeaks of proton pairs assigned to a single parental type (Figure 3). All of the cross-interfacial NOE interactions that have normalized intensities significantly above 0.5 arise from sequential interactions of the amides at the hybrid segment boundaries with the H^α ^or H^β ^of the preceding residue, while the large majority of the less intense crosspeaks arise from long range interactions.

### NMR-directed hybrid rubredoxin structural modeling

The *Cp *rubredoxin (1.2 Å resolution) [29] and N-Met form of *Pf *rubredoxin (1.1 Å resolution) [30] X-ray structures were superimposed with a backbone rmsd of 0.63 Å. The coordinates for residues 1–6, 12–38 and 51–54 from *Cp *rubredoxin were combined with those of residues 7–11 and 39–50 from the *Pf *protein, to yield the metal binding site-swapped *Cp *hybrid. Since three of the four metal-coordinating cysteines are provided by the residue 7–11 and residue 39–50 segments, the position of the metal was derived from the *Pf *rubredoxin structure. The complementary combination yielded the metal binding site-swapped *Pf *hybrid. No additional modifications of these hybrid rubredoxin models were performed.

At each severed mainchain C-N linkage, the interatomic distance is within 0.23 Å of the optimal bond length, and the apparent bond angles at those junctions are all within 7° of ideal geometry. The length of the regenerated Cys 6 sulfur-metal bond is within 0.05 Å of the parental value for both hybrids. The four mainchain-mainchain hydrogen bonds that were interchanged during the formation of the hybrid structures are regenerated with good geometry. The 3.4 Å contacts of Lys 46 C^ε ^with both Phe 30 C^ε ^and Leu 33 C^β ^are the only interfacial nonbonded interactions to have distances less than 0.90 times the sum of the van der Waals radii for the metal binding site-swapped *Pf *A2K rubredoxin model. The Phe 49 H^α^-Thr 5 O distance of 2.1 Å is the only such interaction in the complementary hybrid structure. Analogous model constructions were carried out with several other X-ray structures of *Cp *rubredoxin. The 5RXN [31] Protein Data Bank coordinate file yielded a similar quality of stereochemistry at the interface, while the 1IRO [29], 1FHH [32] and 1FHM [32] coordinate sets each produced an interfacial geometry of substantially lower quality.

### Conformation of the Lys 46 sidechain

**Figure 5:**
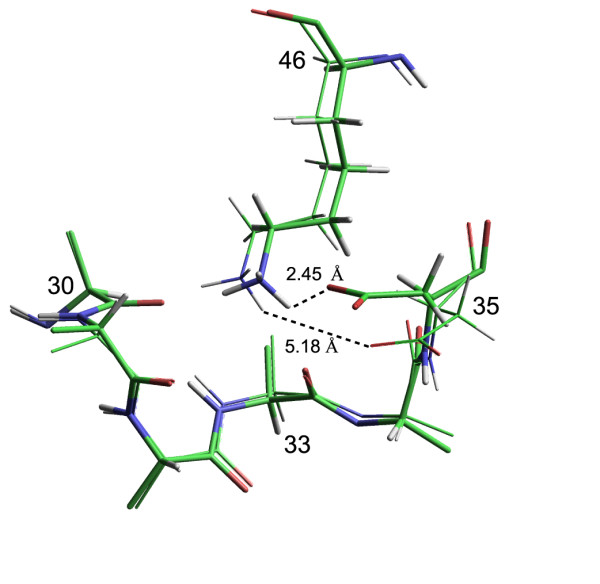
**Interactions of the Lys 46 sidechain in the NMR-derived metal binding site-swapped rubredoxin structures**. As observed in the X-ray crystal structures of both *Cp *and *Pf *rubredoxins, the ε-amino group of Lys 46 hydrogen bonds to the backbone carbonyl oxygens of residues 30 and 33. An additional interaction with the carboxyl sidechain of Asp 35 is observed in the metal binding site-swapped *Cp *rubredoxin (thick-line structure). In contrast, the Asp 35 carboxyl of the metal binding site-swapped *Pf *rubredoxin (thin-line structure) is rotated away toward the aqueous phase, more closely approaching the solvent-exposed Asp 35 H^N^.

Lys 46 is highly conserved throughout the family of rubredoxin sequences. The symmetrical displacement of the Lys 46 H^ε ^chemical shift values from the diagonals in panels A and B of Figure 2 suggests that the local structural environment at the end of this sidechain in each hybrid does not resemble the environment in the parental rubredoxin that is predicted from the Lys 46 mainchain amide chemical shifts, but rather it resembles the environment in the opposite parent. A similar reversed correlation is observed for several Lys 46 sidechain NOE interactions which span the hybridization interface. Figure 5 illustrates the interaction of this sidechain with the backbone segment from Phe 30 to Asp 35 for the NMR-derived structure of each metal binding site-swapped hybrid. As in the parental rubredoxin structures, the ε-amino group of Lys 46 is hydrogen bonded to the mainchain carbonyl oxygens of residues 30 and 33. In the metal binding site-swapped *Cp *rubredoxin model, the sidechain carboxylate group of Asp 35 bonds with the ε-amino group of Lys 46, as seen in the various X-ray structures of *Cp *rubredoxin. In contrast, the Asp 35 sidechain is rotated out toward the solvent in the metal binding site-swapped *Pf *hybrid model (Figure 5), as occurs in the *Pf *rubredoxin X-ray structure. Given the otherwise highly similar environment of the backbone amide of Asp 35 among the structures, this carboxylate reorientation likely accounts for the nearly 0.4 ppm differential shift of the Asp 35 H^N ^resonance (Figure 1). In considering what interactions could give rise to this altered conformation, we note that the van der Waals contact of residue 33 (Ile in *Cp *and Leu in *Pf*) is the only direct interaction of Lys 46 with a residue that differs between the *Cp *and *Pf *sequences.

### 0.79 Å X-ray Analysis of the metal binding site-swapped *Cp* rubredoxin

The metal binding site-swapped *Cp *rubredoxin crystallized in a P2_1 _space group with three protein molecules per asymmetric unit, as earlier observed for a V44A variant of *Cp *rubredoxin [33]. In contrast, the *Cp *rubredoxin structure [29] used in the NMR-derived structure had crystallized in the R3 space group, with a single molecule in the asymmetric unit. Molecular replacement with the V44A *Cp *rubredoxin coordinates was used for initial phasing. Refinement was extended to higher resolution with inclusion of heavy atom anisotropic temperature factors and all hydrogen atoms, to attain a final R (R_free_) factor of 11.2% (12.5%) for all reflections out to 0.79 Å (Table 2). The rmsd values for the backbones of the A, B and C monomers with respect to the original search model were only 0.22 Å, 0.24 Å and 0.27 Å, respectively.

**Table 2:** Data collection, refinement, and final model statistics.

	Metal binding site-swapped *Cp *rubredoxin	Metal binding site-swapped *Pf *A2K rubredoxin
***Data Collection***		
Space group	*P*2_1_	*P*2_1_
Cell dimensions (Å)	*a *= 38.18 Å, *b *= 56.86 Å, *c *= 38.17 Å, β = 112.92°	*a *= 45.26 Å, *b *= 45.84 Å, *c *= 95.08 Å, β = 98.43°
Resolution range (Å)	50–0.79 (0.82–0.79)	50–1.04 (1.08–1.04)
No. of unique reflections	147089	177843
Redundancy	3.3 (1.4)	4.4 (3.2)
Completeness (%)	90.0 (36.7)	95.0 (92.3)
Average *I/σ(I)*	27 (3.7)	37 (2.6)
*R*_*merge *_(%)	5.4 (19.7)	5.7 (58.4)
		
***Refinement***		
Resolution limits (Å)	10–0.79	10–1.04
No. reflections	138138	167333
*R*_*work *_(%) (*I *> 4*σ(I)*)	10.6	11.7
*R *(all data)	11.2	13.9
*R*_*free *_(%) (*I *> 4*σ(I)*)	12.1	15.8
*R*_*free *_(%)	12.5	18.0
No. of molecules per asymmetric unit	3	8
Non-H atoms		
Protein	1300	3433
Zinc	3	8
Acetate	3	0
Ethylene glycol	1	0
Water (full/partial)	251/47	640/46
Average B (Å^2^)		
All atoms	11.6	18.1
Main-chain atoms	7.2	13.7
Side-chain atoms	10.4	18.2
Solvent	22.6	29.0
Geometry		
rmsd bond length (Å)	0.016	0.014
rmsd bond angle (°)	2.2	2.3

**Figure 6:**
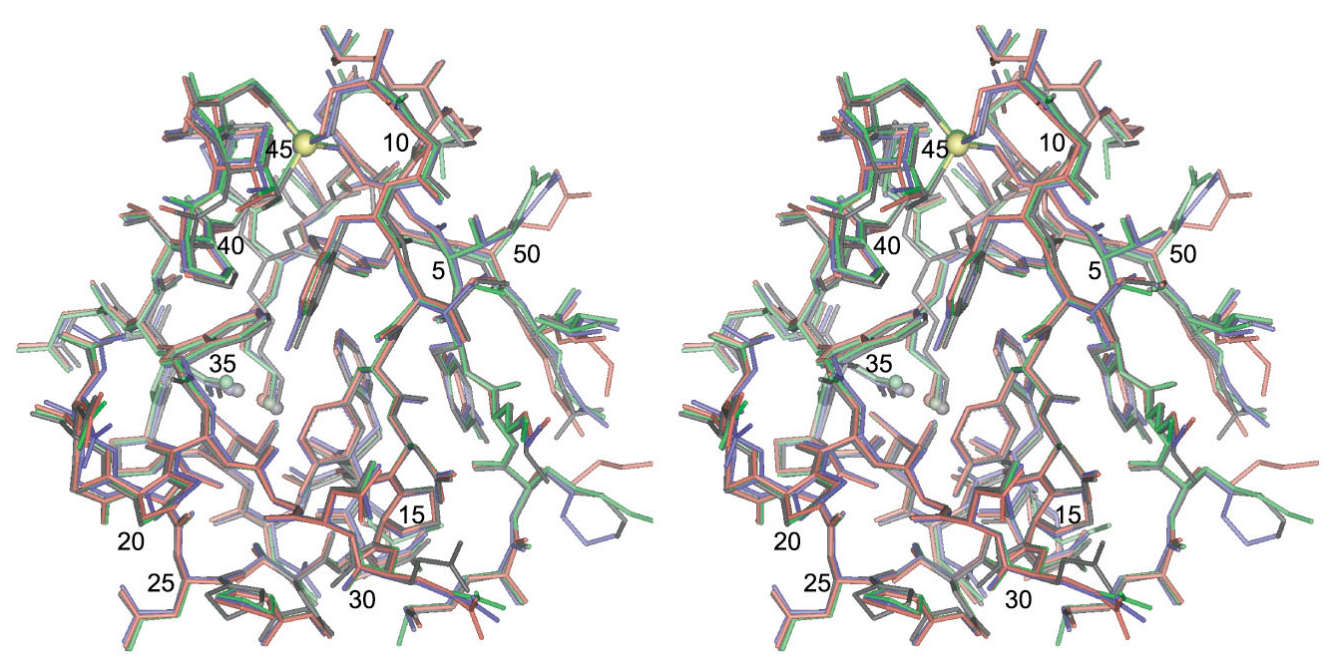
**Superposition of the X-ray and NMR structures of the metal binding site-swapped *Cp *rubredoxin**. Molecules A, B and C of the asymmetric unit in the 0.79 Å resolution X-ray structure are indicated in green, blue and black, respectively, while the NMR-derived structure is indicated in red. The zinc atom is indicated in yellow. The C-terminal residues Glu 53 and Glu 54 were not modeled due to the absence of well-defined density in the electron density map. The Asp 35 O^δ1 ^and Lys 46 N^ζ ^atoms of each structure are highlighted as spheres. For residues exhibiting dual conformations, the more highly populated conformer is displayed.

Figure 6 illustrates the superposition of the three independent molecules of the asymmetric unit along with the NMR-derived structure (red). Molecules A (green) and B (blue) are appreciably more similar to one another than is either of these structures to Molecule C (black). Molecule C diverges most notably from the other three molecules at residues Pro 45, Lys 46 and Ser 47, for which all backbone and sidechain heavy atoms differ by at least 0.5 Å from the superposition with any of the other molecules. Note that the direct interaction between the Lys 46 and Asp 35 sidechains that was predicted in the NMR analysis is indeed observed in the crystal structure.

The average deviations for all sidechain and mainchain heavy atoms in the NMR and X-ray models are given above the diagonal in Table 3 for residues 1 through 52. The analogous calculation for residues 1 through 49 yields a decrease of ~15% in average deviation for the NMR structure, relative to Molecules A (0.317 Å) and B (0.282 Å) in the X-ray structure. The only heavy atoms in the residues 1–49 for which the NMR-derived structure differs from both Molecules A and B by more than 0.5 Å, and furthermore differs from Molecules A and B by more than they differ from each other are: Glu 16 O^ε2^, Ile 41 C^δ^, Ser 47 O^γ^, the carboxyl group of Glu 48 and the C^δ^, C^ε ^and N^ζ ^atoms of Lys 2 and Lys 46. The sidechains of both Ile 41 and Glu 48 exhibit dual conformers in the electron density map.

**Table 3:** Average deviation (Å) of all sidechain and mainchain heavy atoms for the NMR-derived structure and the monomers of the X-ray unit cell for the rubredoxin hybrids.

	NMR^a^	molecule A^a^	molecule B^a^	molecule C^a^	
NMR^b^		**0.383**	**0.327**	**0.487**	NMR^a^
molecule A^b^	0.419		**0.316**	**0.420**	molecule A^a^
molecule B^b^	0.475	0.294		**0.448**	molecule B^a^
molecule C^b^	0.552	0.387	0.354		
molecule D^b^	0.532	0.376	0.418	0.303	
	NMR^b^	molecule A^b^	molecule B^b^	molecule C^b^	

a. Residues 1–52 of the metal binding site-swapped *Cp *rubredoxin (**bold**).

b. Residues 1–52 of the metal binding site-swapped *Pf *A2K rubredoxin.

A clear difference between the NMR and X-ray structures occurs at the Glu 50 sidechain, for which the χ_1 _dihedral angle is 175° in the NMR-derived structure but near +60° in the X-ray structures, despite the fact that the 4.9 Å charge interaction with the Lys 7 sidechain is preserved in both cases. For the metal binding site-swapped *Cp *hybrid and the parental *Pf *A2K rubredoxin, the intraresidue NOE crosspeak values between H^α ^and the degenerate H^β ^resonances of Glu 50 deviates further from the diagonal than any other nonhighlighted NOE in Figure 3C at (0.379, 0.606). The stronger NOE crosspeak for the hybrid protein is consistent with a switch to a +60° χ_1 _angle, for which both β protons are gauche to the H^α^. However, the degeneracy of the Glu 50 H^β ^resonances precluded a clear demonstration of such a conformational shift on the basis of these NOE data alone.

The rubredoxin knuckle structure is observed in nearly all members of the zinc finger superfamily [34]. The metal binding site-swapped *Cp *rubredoxin analysis at 0.79 Å represents the highest resolution Zn^2+^-form protein structure reported to date. Given the marked similarity in Zn^2+ ^and Fe^2+ ^binding site geometries observed for *Cp *rubredoxin [29,32], the present structure may offer a useful model for the reduced active site as well. Regarding crystal lattice interaction analysis, hydrophobin HFBII [35] is currently the only protein in the Protein Data Bank with multiple nonequivalent monomers in the asymmetric unit which has been reported at a resolution exceeding that of this metal binding site-swapped *Cp *rubredoxin structure.

### 1.04 Å X-ray Analysis of the metal binding site-swapped *Pf* A2K rubredoxin

The metal binding site-swapped *Pf *A2K rubredoxin crystallized at pH 5.5 in a P2_1 _space group, with eight molecules per asymmetric unit. In contrast, the P2_1_2_1_2_1 _crystals used to determine the *Pf *rubredoxin structure [30] formed at pH 8.5, with a single molecule in the asymmetric unit. As summarized in Table 2, the refinement for all reflections out to 1.04 Å, utilizing anisotropic B-factors and all hydrogens, yielded a final R (R_free_) factor of 13.9% (18.0%).

The average deviations for all sidechain and mainchain heavy atoms of residues 1–52 in the first four molecules of the asymmetric unit are listed below the diagonal in Table 3. A similar set of values is obtained when molecules E through H are compared among themselves. However, when molecules of the first set (A-D) are compared against those of the second set (E-H), the resultant deviations are approximately 1 Å. The asymmetric unit is arranged as four pairs of dimers. Residues 20–23 from molecules A-D form an asymmetric interaction with the corresponding loop residues of their dimer partners of molecules E-H. All of the first four molecules of the asymmetric unit agree with one another to within less than 0.3 Å for all sidechain and mainchain heavy atoms, when residues 20–26 are excluded. It should be noted that the truncated rubredoxin from *Desulfovibrio desulfuricans *[36] demonstrates that residues 20 to 26 can be deleted without the induction of substantial changes in the rest of the protein structure. Despite the differences in sequence, space group and pH conditions, the backbone coordinates of molecule A differ from those of the original search model by an rmsd value of only 0.22 Å, with modestly larger values for Molecules B, C and D. In contrast, the deviations from the backbone coordinates of the search model were approximately 0.6 Å larger for molecules E-H.

For Molecules A to D of the metal binding site-swapped *Pf *A2K rubredoxin, sidechains exhibiting dual conformations are Ser 25, Val 44 and Gln 48. The interactions of the three charged residues Lys 29, Glu 31 and Glu 32 vary substantially among the molecules of the asymmetric unit, with the Lys 29 sidechain exhibiting weak electron density. Relative to the X-ray structure, the NMR-derived structure adopts a differing χ_1 _dihedral angle for Asp 47 and χ_2 _for Glu 50. If 18 sidechain atoms from these five charged residues and Lys 3 are removed from the analysis (out of 401 atoms), the average deviation for all sidechain and mainchain heavy atoms between the NMR-derived structure and Molecule A of the X-ray structure decreases to less than 0.3 Å. The Asp 35 sidechain of the metal binding site-swapped *Pf *A2K rubredoxin X-ray structure is rotated out toward the solvent phase, away from the Lys 46 N^ζ ^atom, consistent with the findings of the NMR analysis.

## Discussion and Conclusion

Interchange of the seven nonconserved residues in the metal binding site region between the *Cp *and *Pf *A2K rubredoxins yields two complementary hybrid proteins that are accurately represented as a sum of segments from the parental structures. These "cut-and-paste" models match the ultra-high resolution X-ray models to within the structural variability exhibited by the crystallographically nonequivalent molecules in the unit cell. Both of the parental rubredoxin X-ray structures that were used to derive the NMR structures differ with respect to space group and internal packing geometries from those of the crystals used in the hybrid rubredoxin X-ray analyses, indicating that lattice interactions have given rise to minimal deviations among the derived structures. The striking consistency of both the chemical shift and NOE data, among the hybrid and parental rubredoxins, indicates that structural additivity applies to the solution phase as well. Nearly all of the NOE crosspeaks exhibited differential peak heights consistent with the presence of equivalent local interactions to within, at most, 2-fold of the raw spectral noise level. Exceptions to this NOE peak height correlation were generally found to be explicable in terms of differential local dynamics.

The metal binding site-swapped hybrids were designed on the basis of the potential for specific structural segments of the parental *Pf *and *Cp *rubredoxins to form hybridization interfaces in which each of the pairwise interactions across the interfaces in the hybrid proteins would have a 1-to-1 correspondence with an equivalent interaction in the parental protein structures. Within the statistical limits of the X-ray and NMR experimental data, this structural additivity is satisfied. These similarities in structure suggest that the energetics of each interaction in the native state is likely to be similar for the corresponding hybrid and parental rubredoxins. The observed additivity in thermodynamic stability may reflect the fact that the 1-to-1 correspondence between equivalent interactions can be anticipated to apply to the unfolded state as well [13]. The symmetric pattern of variation exhibited in the hydrogen exchange for these rubredoxins [14] indicates that, for at least a subset of the conformational excited states, additivity in the energetics for the hybrid and parental proteins is preserved.

The fact that the metal binding site-swapped hybrids strikingly preserve "cut-and-paste" structures from the parental *Cp *and *Pf *rubredoxins in both crystal and solution does not formally demonstrate that the hybrid structures are the same in those two states. However, given that the NMR-derived structures are based on crystals having substantially different lattice packing and pH environments, their congruence with the ultra-high resolution X-ray structures of the hybrid rubredoxins renders unclear the physical basis upon which a substantial deviation between solution and crystal structure would arise. The present study provides no support for the interpretation that the 1.6 Å average difference in backbone atom positions that has been observed between corresponding X-ray and NMR-derived structures [21] is primarily a manifestation of differences in conformation between the protein in solution and in the crystal. In this regard, it should be noted that a recent analysis of 148 proteins concluded that crystal packing plays a minor role in the observed differences between the homologous crystal and NMR structures [37].

More than 500 NMR structure determinations have been recalculated and refined by restrained molecular dynamics in a hydrated environment, following a standard protocol utilizing both CNS and CYANA [20]. Application of more physically realistic energy potentials substantially improved the quality of the stereochemistry in the resultant NMR structures, as analyzed by PROCHECK and WHAT_CHECK. However, the average rmsd value for the ensemble of well-ordered backbone atoms increased by nearly 2-fold (i.e. from 0.6 Å to 1.1 Å). A similar increase in rmsd was observed when that rmsd value was maximized for the set of structures compatible with a given set of NMR restraints, through a process of conformational resampling [38]. Among the more than 500 NMR structures analyzed, analogous X-ray coordinates were available for 26 proteins [20]. For these 26 cases, both the original and the recalculated NMR structures are equally distant from the corresponding X-ray structure. Hence, the systematic deviations from the analogous X-ray determinations do not appear to arise from how the structures are derived from the reported NOE distance restraints, but rather they primarily arise from the NOE distance restraints themselves.

Potential insight into the discrepancy between homologous X-ray and NMR structure determinations can be gained by noting that X-ray protein structure analyses carry out an iterative mapping of the predicted electron densities onto the experimental diffraction intensities. In contrast, NMR analyses do not refine their progression of model structures directly against the observed NOE data, but rather against a set of derived distance restraints. The setting of NOE-based distance restraints to the maximum range of physically plausible values results in a large dispersion of compatible structures. In response, numerous approaches have been proposed for tightening the assumed range of upper and lower distance bounds [39-43]. These derived distance restraints are then commonly treated as dependent variables for the progression of model structures, so that discordant values in the restraint list are edited during the course of the structure analysis by readjustment of distance boundaries [44] and by a "cherry-picking" of the set of crosspeaks [45] that are consistent with the current model. More recently, algorithms [46,47] have been proposed for iterative readjustment of the NOE-based restraints, such that both the optimal interproton target distance and the weighting of the individual experimental measurements are scaled according to the degree of disagreement with the progression of structural models. As a result of such iterative readjustments, the NOE distance restraint list ceases to provide an independent test of that structural model.

Refinement of the solution structure against the experimental NOE intensities can be carried out using relaxation matrix analysis to calculate the peak intensities predicted for a given structural model. For technical reasons, this is most often performed via recalculation of the distance boundaries based on relaxation matrix calculations for the current structural model. Although earlier concerns over the computational demands of this approach have abated with recent advances in computer technology, significant issues remain as to how best to incorporate conformational and chemical exchange effects into the calculations [48-53]. In parallel, progress in the computational analysis of chemical shifts [54-56] may soon enable the exquisite sensitivity to local structure exhibited by these data to be more robustly integrated into the structural refinement protocols.

Quantitative NOE and chemical shift difference analysis might be most effectively applied to the characterization of moderately divergent structural homologs. The comparative modeling component of the recent CASP6 study analyzed 27 proteins for which BLAST [57] sequence alignment had identified a homologous X-ray structure [58]. The median fit to the backbone between the modeled and experimentally determined target structures was 1.7 Å, equivalent to the average difference between NMR and X-ray structure determinations for identical proteins [21]. Given this apparent statistical equivalence, it is of considerable importance to demonstrate what increased structural information is gained from a de novo solution structural analysis of a given target protein as compared to that obtained from a molecular modeling analysis based on the X-ray structure of a moderately divergent protein homolog. Further developments in relaxation matrix and chemical shift analysis methods might be efficiently optimized by applying these approaches to such pairs of homologous proteins. By carrying out parallel measurements on the two protein homologs, differential NMR analysis could provide insight into structural details that are not obtained by the independent analysis of each protein via conventional NMR structure determinations.

## Methods

### NMR measurements and processing

Zn^2+^-form ^15^N-labeled samples of *Cp *rubredoxin, *Pf *A2K rubredoxin and the complementary metal binding site-swapped hybrids were prepared as previously described [13,23]. The 2–3 mM protein samples were serially dialyzed against a buffer containing 100 mM sodium chloride, 20 mM sodium phosphate and 20 mM boric acid at pH 6.00. Data collection was carried out on a Bruker DRX 500 at 23°C. 2D COSY and 2D ^15^N-suppressed ^1^H-^1^H NOESY and TOCSY spectra were collected with Watergate suppression [59]. 2D ^1^H-{^15^N}-^1^H TOCSY and 3D ^1^H-^15^N-^1^H NOESY spectra were obtained utilizing FHSQC detection [60]. Mix times of 200 ms and 83 ms were used for the NOESY and TOCSY spectra, respectively. The NMR data were processed with the FELIX software package (Accelerys, San Diego, CA). NOESY crosspeak heights and volumes were determined using automated peak selection, followed by manual editing in NMRView [61]. Relaxation-compensated CLEANEX-PM measurements [25,26] on these samples were conducted as previously described [62].

The analysis utilized crosspeaks between pairs of protons separated by at least one flexible dihedral angle. All retained crosspeaks exhibited no significant overlap or other visible spectral distortion and lay within 0.015 ppm (7.5 Hz) and 0.2 ppm (10 Hz) of the assigned chemical shift in the ^1^H dimensions and in the ^15^N dimension, respectively. The *Cp *and N-Met *Pf *rubredoxin X-ray structures were used to exclude all crosspeaks that could potentially arise from more than one pair of protons that are separated by less than 7.5 Å for NOE crosspeaks between amide and methyl protons and less than 6 Å in all other cases. The well resolved 3D NOE crosspeaks analyzed in this study include 83% and 87%, respectively, of all proton pairs predicted to lie within 5 Å of each other in the NMR-derived structures of the metal binding swapped hybrids of the *Cp *and *Pf *A2K rubredoxins. Over half of the remainder of predicted crosspeaks corresponds to proton pairs that are separated by at least 4.5 Å. The completeness of the set of analyzed crosspeaks from the 2D NOE spectra is significantly less, due to the much higher fraction of visibly overlapped resonances.

The 3D NOE intensities of the *Pf *A2K spectrum were normalized to the peak height of the Trp 37 H^δ1^-H^ε1 ^crosspeak. The 3D NOE crosspeak intensities from the *Cp *and the hybrid rubredoxins were then correlated with the analogous *Pf *A2K intensities, and the regression line was scaled to unity, so as to adjust for differences in sample concentration and data acquisition reproducibility. The analogous sorting and scaling procedure was applied to the ^15^N-suppressed 2D NOE intensities, with the *Pf *A2K Trp 37 H^ε3^-H^ζ3 ^crosspeak used as internal intensity reference.

### NMR-directed hybrid rubredoxin structural modeling

The coordinates for *Cp *rubredoxin [PDB ID:1IRN] [29] and N-Met *Pf *rubredoxin [PDB ID:1BQ8] [30] were superimposed with LSQMAN [63], using a set of 341 structurally conserved [12] heavy atoms. Hydrogen atoms were added with the program Reduce [64]. Atoms for residues 7–11, 39–50, and the metal were interchanged between the *Cp *and N-Met *Pf *coordinate sets to generate models of the complementary metal binding site-swapped hybrids. The Lys 2 sidechain of the metal binding site-swapped *Pf *A2K hybrid was not modeled. Bond lengths and angles were analyzed with Procheck [18], and nonbonded contacts were calculated with Macromodel [65].

### Crystallization

Zn^2+^-form protein samples, equilibrated at pH 6.0, were dialyzed against deionized water and then concentrated to 20 mg/ml for the metal binding site-swapped *Pf *A2K rubredoxin and to 40 mg/ml for the metal binding site-swapped *Cp *rubredoxin. Crystals were grown at room temperature in hanging drops, by mixing 2 μl of protein solution with an equal volume of reservoir solution. The metal binding site-swapped *Cp *rubredoxin was crystallized in a solution containing 48% saturated ammonium sulfate, 3% ethylene glycol and 0.1 M sodium acetate at pH 4.5, while the metal binding site-swapped *Pf *A2K rubredoxin was crystallized in a reservoir solution containing 62% saturated ammonium sulfate and 0.1 M sodium citrate at pH 5.5.

### X-ray data collection, structure determination and refinement

The metal binding site-swapped *Cp *rubredoxin crystals (0.3 * 0.3 * 0.4 mm) were transferred to a reservoir solution containing 25% glycerol, flash-cooled under a nitrogen stream at 100K and then stored in liquid nitrogen. Prior to flash cooling, the metal binding site-swapped *Pf *A2K rubredoxin crystals (0.6 * 0.6 * 0.2 mm) were transferred through a series of reservoir solutions with successive 5% increases in ammonium sulfate, to a final concentration of 85% saturated ammonium sulfate to enhance stability during low temperature data collection. To obtain ultrahigh resolution on the synchrotron X-ray beamline X25 of the National Synchrotron Light Source (NSLS) at Brookhaven National Laboratory, collection was carried out at a wavelength of 0.8577 Å on a ADSC Q315 CCD detector with an oscillation angle and range of 1° and 160°, respectively. Two sets of diffraction data were collected for each crystal, with a different exposure time for each set: 1 sec (to 1.2 Å) and 10 sec (to 0.79 Å) for the metal binding site-swapped *Cp *rubredoxin crystal, and 3 sec (to 1.4 Å) and 15 sec (to 1.04 Å) for the metal binding site-swapped *Pf *A2K rubredoxin crystal. All of the data were processed and scaled using HKL2000 [66]. Although the R_merge _value for the highest shell of the metal binding site-swapped *Pf *A2K rubredoxin was elevated, the nearly 20,000 reflections in this shell were retained due to their good redundancy (3.2) and a high average (I/σI) (2.6).

The metal binding site-swapped *Cp *rubredoxin crystal is isomorphous with that of the *Cp *rubredoxin V44A mutant [PDB ID:1C09] [33]. Accordingly, the latter structure was used directly for the initial phasing. The structure of the metal binding site-swapped *Pf *A2K rubredoxin was solved using the PHASER program[67], with the 1.1 Å crystal structure of Met-terminal *Pf *rubredoxin [PDB ID:1BQ8] [30] as the search model.

Structural refinement for both hybrid rubredoxin crystals was initially carried out at 2.0 Å resolution using CNS v 1.0 [68], with 5% of the data set aside as the test data for the R_free _cross-validation. After initial rigid-body refinement, iterative cycles of positional and temperature factor refinement were carried out, interspersed with model rebuilding into σ_A_-weighted (F_o_-F_c_) and (2F_o_-F_c_) electron density maps using the program TURBO FRODO [69]. At later stages of the structural refinements, the program suite SHELX97 [70] was used, with inclusion of all reflection data out to 1.04 Å for the metal binding site-swapped *Pf *A2K rubredoxin and out to 0.79 Å for the metal binding site-swapped *Cp *rubredoxin. Each round of refinement consisted of 10–20 cycles of conjugate-gradient least-squares refinement. Water molecules automatically introduced by SHELXH were evaluated manually after each round of refinement. The disorder of main-chain and side-chain atoms was modeled. Anisotropic atomic displacement parameters for all non-H atoms were introduced, resulting in a significant decrease of *R*_free_. After modeling of solvent disorder, the H atoms were added and refined with isotropic temperature factors set by default to 1.2 or 1.5 times the *B *factor of the bound atom. The *Cp *and *Pf *A2K rubredoxin hybrids exhibited dihedral angle deviations of 26.5° and 26.3°, respectively, while the corresponding improper torsion angle values were 1.8° and 1.6°. Atomic coordinates have been deposited in the Protein Data Bank as entries 2PVE (*Cp *rubredoxin hybrid) and 2PVX (*Pf *A2K rubredoxin hybrid).

## Abbreviations

NMR, nuclear magnetic resonance; NOE, nuclear Overhauser enhancement; *Cp*, *Clostridium pasteurianum*; *Pf*, *Pyrococcus furiosus*; rmsd, root mean square deviation.

## Authors' contributions

DML and GH conceived the project and performed the NMR measurements. JSA, DML and GH carried out the analysis of the NMR data. YG conducted the crystallization experiments. HL obtained the diffraction data. LW and HL carried out the X-ray refinement. DML, JSA, HL and GH contributed to the writing of the manuscript.
